# Virulence of Marburg Virus Angola Compared to Mt. Elgon (Musoke) in Macaques: A Pooled Survival Analysis

**DOI:** 10.3390/v10110658

**Published:** 2018-11-21

**Authors:** Paul W. Blair, Maryam Keshtkar-Jahromi, Kevin J. Psoter, Ronald B. Reisler, Travis K. Warren, Sara C. Johnston, Arthur J. Goff, Lydia G. Downey, Sina Bavari, Anthony P. Cardile

**Affiliations:** 1 Division of Infectious Diseases, Johns Hopkins University School of Medicine, Baltimore, MD 21205, USA; pblair6@jhmi.edu (P.W.B.); maryam.keshtkar@jhmi.edu (M.K.-J.); 2 Department of Pediatrics, Johns Hopkins University School of Medicine, Baltimore, MD 21224, USA; kpsoter1@jhu.edu; 3 United States Army Medical Research Institute of Infectious Diseases, Fort Detrick, Frederick, MD 21702, USA; ronald.b.reisler.ctr@mail.mil (R.B.R.); travis.k.warren.ctr@mail.mil (T.K.W.); sara.c.johnston2.civ@mail.mil (S.C.J.); arthur.j.goff.civ@mail.mil (A.J.G.); ldybg88@gmail.com (L.G.D.); sina.bavari.civ@mail.mil (S.B.)

**Keywords:** Marburg virus disease, hemorrhagic fevers, viral, models, animal, filoviridae, mononegavirales, marburgvirus, Angola, Musoke

## Abstract

Angola variant (MARV/Ang) has replaced Mt. Elgon variant Musoke isolate (MARV/MtE-Mus) as the consensus standard variant for Marburg virus research and is regarded as causing a more aggressive phenotype of disease in animal models; however, there is a dearth of published evidence supporting the higher virulence of MARV/Ang. In this retrospective study, we used data pooled from eight separate studies in nonhuman primates experimentally exposed with either 1000 pfu intramuscular (IM) MARV/Ang or MARV/MtE-Mus between 2012 and 2017 at the United States Army Medical Research Institute of Infectious Diseases (USAMRIID). Multivariable Cox proportional hazards regression was used to evaluate the association of variant type with time to death, the development of anorexia, rash, viremia, and 10 select clinical laboratory values. A total of 47 cynomolgus monkeys were included, of which 18 were exposed to MARV/Ang in three separate studies and 29 to MARV/MtE-Mus in five studies. Following universally fatal Marburg virus exposure, compared to MARV/MtE-Mus, MARV/Ang was associated with an increased risk of death (HR = 22.10; 95% CI: 7.08, 68.93), rash (HR = 5.87; 95% CI: 2.76, 12.51) and loss of appetite (HR = 35.10; 95% CI: 7.60, 162.18). Our data demonstrate an increased virulence of MARV/Ang compared to MARV/MtE-Mus variant in the 1000 pfu IM cynomolgus macaque model.

## 1. Introduction

Marburg virus is a deadly filovirus that is in the same family as the widely recognized Ebola virus. Marburg virus disease (caused by Marburg virus; order *Mononegavirales*: family *Filoviridae*) is less well known and less studied than Ebola virus disease [1,2]. However, Marburg virus causes an equal or higher case fatality rate (CFR) in humans than Ebola [3], and the discovery of Marburg virus predated that of Ebola virus [4]. In 1967, Marburg virus was first identified in Marburg, Germany when workers at a research laboratory were infected from non-human primate animal products. Since 1967, sporadic outbreaks of Marburg disease have occurred in central Africa, primarily in areas where the suspected reservoir, the Egyptian roussette (*Rousettus aegyptiacus*), is known to reside [5]. Between 1998 and 2000, there was an 83% CFR among the 154 cases in the Democratic Republic of the Congo, and between 2004 and 2005 there was a 88% CFR in Angola among 375 cases [6,7]. However, Marburg virus outbreaks have also occurred outside of this endemic area leading to severe disease and death in locations worldwide [5]. Marburg virus was introduced to the United States and the Netherlands in 2008 after two independent exposures of tourists visiting a cave in Uganda [8,9]. Additionally, sporadic cases have continued to occur with two recent fatal human cases recently in Uganda in November 2017 [10]. Despite the potential for outbreak propagation which could result in a high human cost, there are no approved vaccines or therapeutics for the prevention or treatment of Marburg virus disease (MVD). 

Differences in the clinical pathogenesis of MVD have strong implications for both the interpretation of research using different strains or variants and for the development of medical countermeasures. Of note, CFRs in human outbreaks of Marburg virus disease have varied greatly, from 27 to 88% [3]. MARV/Ang, which has become the consensus standard, was originally collected in 2005 in Uige, Angola from a fatal human case and was associated with the highest human CFR to date of a major MVD outbreak (88%; 158 confirmed cases, 329 deaths/375 total cases) [3,7]. However, clinical data is limited from that outbreak [11].

Marburg Angola variant (MARV/Ang) replaced Marburg Mt. Elgon variant (MARV/MtE-Mus) as the commonly referred consensus standard. MARV/MtE-Mus, also commonly referred to as “Marburg Musoke” or “MARV/Mus,” was collected in 1980 from a Kenyan survivor who was a physician [12]. Dr. Musoke contracted Marburg virus from a fatal case (1 death/2 total cases) [13]. No other cases were detected during that outbreak, and conclusions about the virulence of that variant in humans are therefore limited [14]. It has also been suggested that variability in virulence as measured by case fatality rates is in part due to differences in supportive care between low and high-resource settings [3]. That being said, the manifestations of the MARV/Ang have also been described to be more severe in nonhuman primates (NHPs) than MARV/MtE-Mus by experts in the field [15]. In one historical comparison, the mean time to death of four NHPs exposed to MARV/Ang by different routes was 7.8 days while the mean time to death of 11 historical NHPs exposed to MARV/MtE-Mus by different routes was 9.8 days [16,17]. However, despite calls to study these differences further, there have not been more robust analyses to support these assertions [14].

Despite limited supporting data, assertions that MARV/Ang had increased lethality largely contributed to it becoming the consensus research standard for MARV studies [14,15,18,19]. Distinguishing the differences in virulence between MARV/Ang and MARV/MtE-Mus in nonhuman primates is important since we rely on non-human primate research for the development and approval of therapeutics and vaccines for Marburg virus. The US Food and Drug Administration (FDA) Animal Rule guidance supports product development via animal studies and phase I clinical trials, because it is unethical and infeasible to perform phase II or III clinical trials with highly lethal and rare pathogens such as Marburg virus [20]. Because the interpretation of study results may differ depending on the expected severity of disease in an animal model, it is necessary to directly compare the natural history of disease between variants to inform the evaluation of novel therapeutics or vaccines [21]. Herein, we sought to compare clinical outcomes between NHPs experimentally infected with 1000 pfu IM of either MARV/Ang or MARV/MtE-Mus. We hypothesized that the MARV/Ang would be associated with an increased risk of death and earlier manifestations of disease than MARV/MtE-Mus.

## 2. Materials and Methods 

### 2.1. Aggregation of Data

Marburg virus (MARV) Angola variant was originally isolated from a fatal case in an 8-month old infant (Marburg virus/H. sapiens-tc/ANG/2005/Angola-1379v) which had been provided to USAMRIID by the CDC and had undergone a total of three passages in Vero cells [22]. Marburg virus (MARV) Musoke isolate was originally isolated from an adult survivor (Marburg virus/H.sapiens-tc/KEN/1980/Mt. Elgon-Musoke) and human serum that was obtained from the CDC from this survivor and had undergone six passages in Vero cells at USAMRIID [13,23]. Between 2012 and 2017, eight experimental studies using the same institutional standard operating procedures were conducted in biosafety level 4 laboratories at the U.S. Army Medical Research Institute of Infectious Diseases. In these studies, crab-eating (cynomolgus) macaques (*Macaca fascicularis*) were exposed with a standardized target 1000 plaque forming units (pfu) of Marburg virus (MARV/Ang or MARV/MtE-Mus) administered intramuscularly [24,25,26]. The same seed stocks of virus were used for experiments that used MARV/Ang, and the same seed stocks were used for experiments that used Mt. Elgon variant. Mt. Elgon variant was utilized in studies conducted in 2012 while MARV/Ang was administered from 2012 to 2017. From these studies, we pooled data collected from animals that did not receive experimental agents (i.e., control animals) and were monitored under standard conditions after infection. Detailed methodology describing the exposure of NHPs to Marburg virus has been previously described [24]. The study population included 25 male and 22 female NHPs, with 18 NHPs administered MARV/Ang as part of three separate studies, and 29 administered MARV/MtE-Mus as part of five studies.

NHPs were followed from the day of exposure until death. As part of each study, NHPs were monitored at least twice per day, which included the serial collection of clinical observations. Blood was collected for laboratory analysis immediately prior to exposure on day 0 (baseline) with at least one blood collection during follow-up and prior to death. There was heterogeneity between studies in laboratory monitoring during follow-up due to protocol differences (Supplemental Table S1). The Piccolo (Abaxis, Abaxis, Union City, CA, USA) or the Vitros 350 Chemistry System (Ortho Clinical Diagnostics, Raritan, NJ, USA) were used to analyze serum chemistries for complete metabolic panels. The Hemavet 950 FS (Drew Scientific, Waterbury, CT) or the Advia 120 Hematology Analyzer (Siemens, Tarrytown, NY, USA) were used to determine complete blood counts. A Sysmex CA-1500 (Siemens) was used for coagulation variables [24,27]. These panels collectively provided results for 46 clinical laboratory parameters. Viral RNA copy numbers were determined using quantitative real-time PCR (qRT-PCR) [27,28].

### 2.2. Ethics Statement

Research was conducted under an Institutional Animal Care and Use Committee (IACUC) approved protocol in compliance with the Animal Welfare Act, PHS Policy, and other Federal statutes and regulations relating to animals and experiments involving animals. The facility where this research was conducted is accredited by the Association for Assessment and Accreditation of Laboratory Animal Care International (AAALAC) and adheres to principles stated in the Guide for the Care and Use of Laboratory Animals, National Research Council, 2011 [29]. The NHPs received oral hydration, food, and environmental enrichment per standard operating procedures and received pain medication as needed. The NHPs were euthanized if they met standardized euthanasia criteria as previously described [24,30].

### 2.3. Analysis

The primary outcome of interest was the time to death post-exposure between variants. Secondary outcomes included the time to development of anorexia and time to development of rash, which are routinely collected clinical observations. Time of death was defined as the time of euthanasia after meeting a pre-defined endpoint or the time found deceased in cage. Time to anorexia was defined as time to any reduction of biscuit intake recorded during observations, and time to development of rash was defined as the time to the development of any rash noted on observations. Secondary outcomes were chosen to represent the onset of clinical disease. These outcomes were chosen due to the low risk of misclassification, almost uniform occurrence, and uniform collection. Exploratory analyses included time to viremia, anemia, leukocytosis, C-reactive protein (CRP) elevation, alanine transaminase (ALT) elevation, aspartate transaminase (AST) elevation, creatinine elevation, fibrinogen decrease, activated partial thromboplastin time (aPTT) elevation, prothrombin time (PT) elevation, and thrombocytopenia. Viremia was defined as 5.12 Log_10_ genomic equivalents (ge)/mL (as determined by qRT-PCR) and represents a lower level of quantification for the MARV/MtE-Mus. High viremia was defined as being greater than the median observed viremia of 6.34 Log_10_ ge/mL. Clinical parameter endpoints were based upon previously described reference ranges [31,32] and were defined as ≥2× the upper limit of normal for parameters expected to increase (e.g. AST) or ≤lower limit of normal for parameters expected to decrease (e.g. fibrinogen) (Supplementary Table S2).

The time to each outcome and laboratory values over time were summarized. Baseline clinical characteristics were compared using Student t-tests with unequal variances. Median laboratory values were plotted over time by variant to visually explore the data. For each variant, Kaplan–Meier plots were used to describe the probability of occurrence of each event over time and were compared using a logrank test. The association of variant type with time to each outcome was evaluated using unadjusted Cox proportional hazards regression and multivariable models which included adjustment for sex, age, and baseline weight. For these models, NHPs entered risk sets at the time of virus exposure and were followed until the event was observed or were censored at time of death for secondary and exploratory endpoints. For laboratory endpoints, NHPs were censored on the day of the last recorded measurement for those in which the event was not recorded. Death was recorded in hours post-exposure, whereas each of the other outcomes were recorded day post-exposure. Results of regression models are presented as hazard ratios (HR) with corresponding 95% confidence intervals (CI). To account for multiple comparisons a Bonferroni corrected P value of 0.0036 was used for primary, secondary, and exploratory endpoints. All analyses were performed using Stata version 15.0 (StataCorp LLC, College Station, TX, USA). 

## 3. Results

### 3.1. Baseline Differences

A total of 47 NHPs were included, of which 18 NHPs were exposed to MARV/Ang and 29 NHPs were exposed to MARV/MtE-Mus. Overall, 53.2% of NHPs were male and were on average 5.63 years old (SD = 1.07) and weighed 4.93 kg (SD = 1.40) (Table 1). In general, the characteristics of NHPs were similar between those exposed to MARV/Ang and MARV/MtE-Mus (Table 1); however, NHPs exposed to MARV/Ang were older (6.14 years; SD 1.12) than those exposed to MARV/MtE-Mus (4.81 years; SD 0.91; *p*-value = 0.008). 

### 3.2. Time to Event Analyses

Following Marburg virus exposure, all NHPs died, and most NHPs experienced anorexia (96%) and rash (94%). Irrespective of the variant, rash was preceded by decreased food consumption by 1.1 days (SD 1.9) on average. Table 2 describes the median time to each outcome overall (both variants combined) and by variant. Overall, the median time to death was 9.0 days (25th, 75th percentiles: 9.0, 10.0). The median time to death for MARV/Ang-infected NHPs was 8.1 days (25th, 75th percentiles: 7.9, 8.9), whereas MARV/MtE-Mus-infected NHPs survived for a median of 10.0 days (25th, 75th percentiles: 9.0, 10.9). Overall, the median time to anorexia was 7.0 days (25th, 75th percentiles: 6.0, 8.0). However, the median time to anorexia was earlier at 6.0 days (25th, 75th percentiles: 6.0, 7.0) during MARV/Ang infection compared to 8.0 days (25th, 75th percentiles: 8.0, 19.0) following MARV/MtE-Mus infection. The overall median time to rash was 9.00 days (25th, 75th percentiles: 7.0, 9.0). The median time to rash was earlier during MARV/Ang infection at 7.0 days (25th, 75th percentiles: 6.0, 7.0) compared to 9.0 days (25th, 75th percentiles: 9.0, 10.0) during MARV/MtE-Mus infection.

**Figure 1:**
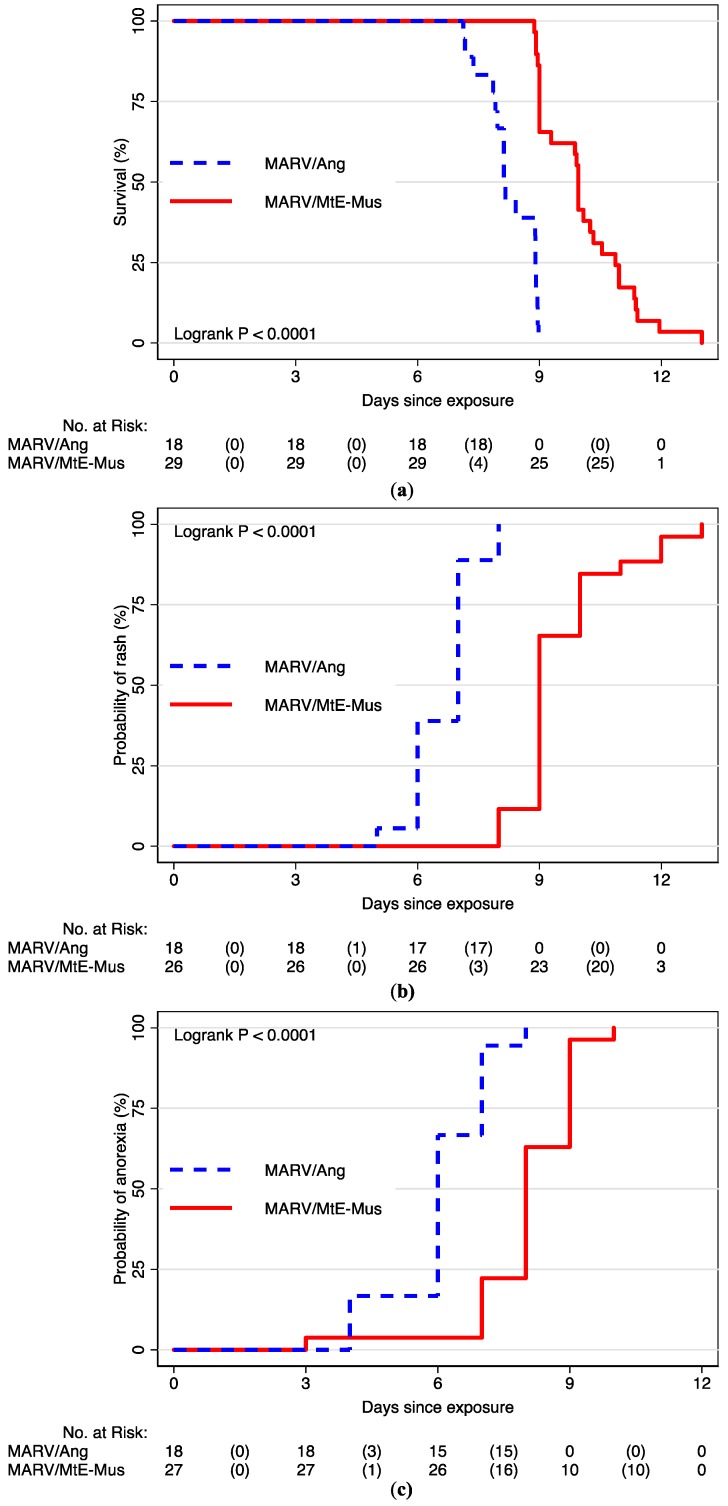
Kaplan–Meier survival curves in cynomolgus macaques by variant for (**a**) survival; (**b**) time to anorexia; and (**c**) time to rash.

MARV/Ang was associated with a statistically significant increased risk of death (HR = 22.10; 95% CI: 7.08, 68.93), development of rash (HR = 5.87; 95% CI: 2.76, 12.51), and anorexia (HR = 35.10; 95% CI: 7.60, 162.18) (Figure 1; Table 3). Of note, when comparing time to death after the onset of anorexia, death occurred at 2.21 days after MARV/Ang exposure and 1.73 days after MARV/MtE-Mus exposure (*p* = 0.187). After anorexia, there was no difference in risk of death between variant (N = 41; HR = 0.71; 95% CI: 0.37, 1.36).

### 3.3. Viremia

**Figure 2:**
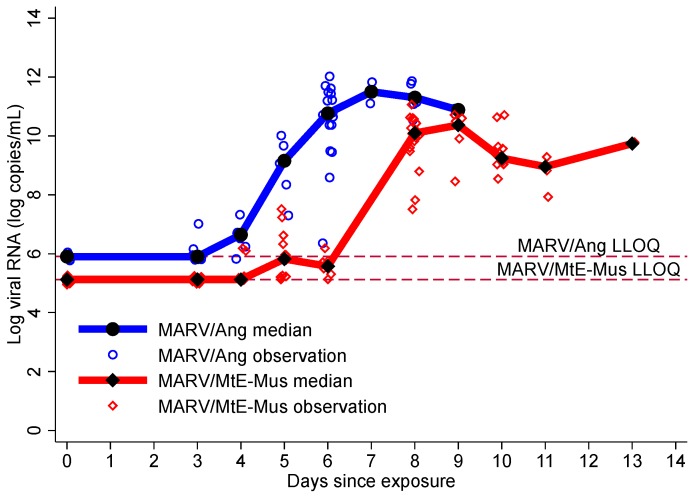
Log viral RNA by variant. The lower level of quantitation (LLOQ) for MARV/Ang was 5.903 log copies/mL and the LLOQ for MARV/MtE-Mus was 5.12 log copies/mL. Therefore, 5.12 log copies/mL was used as a common endpoint for viremia for both variants.

Viremia in both strains combined occurred at a median of 6.0 days and was recorded earlier after MARV/Ang exposure at a median of 4.0 days (25th, 75th percentiles: 3.0, 6.0 days) compared to 6.0 days (25th, 75th percentiles: 5.0, 8.0 days) following MARV/MtE-Mus exposure (Table 2). Following MARV/Ang exposure, the highest median viral load was observed on day 7 at 11.50 log copies/mL, (25th, 75th percentiles: 11.10, 11.90), while the peak MARV/MtE-Mus viral load was observed on day 9 (10.37 log copies/mL; 25th, 75th percentiles: 9.85, 10.57) (Figure 2). Compared to MARV/MtE-Mus, the risk of viremia was higher for MARV/Ang, but this difference was not statistically significant (HR 1.51; 95% CI: 0.69, 3.30; *p*-value 0.306). MARV/Ang exposure was also associated with an increased risk of high viremia, and there was a trend towards significance (HR 3.75; 95% CI: 1.53, 9.18; *p*-value = 0.004).

### 3.4. Clinical Laboratory Parameters

#### 3.4.1. Immune System Activation and Anemia

**Figure 3:**
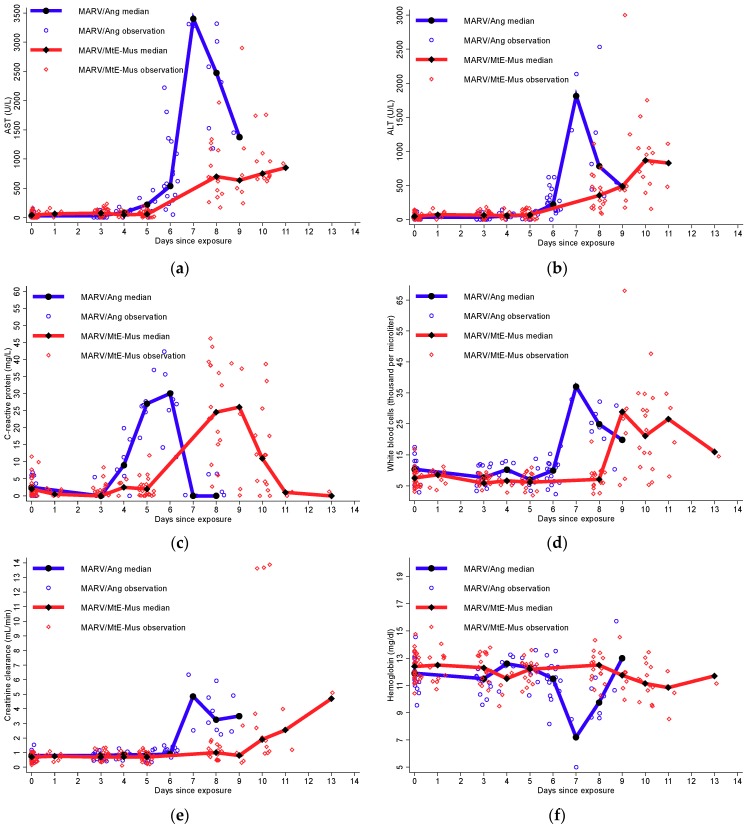
Median and individual laboratory values over time by variant for (**a**) AST (U/L); (**b**) ALT (U/L); (**c**) CRP (mg/L); (**d**) white blood count (thousand per microliter); (**e**) creatinine clearance (mL/min); and (**f**) hemoglobin (mg/dL). Note: ALT, alanine transaminase; AST, aspartate transaminase; CRP, C-reactive protein; WBC, white blood count.

Median markers of inflammation were plotted by variant over time (Figure 3). Laboratory values were available on various days post-exposure, but only days with measurements which included both variants were used to explore the data. At day 5, the median serum CRP was higher during MARV/Ang infection (n = 6) at 27.00 mg/dL (25th, 75th percentiles: 23.00, 30.00) than during MARV/MtE-Mus infection (n = 22) at 2.00 mg/dL (25th, 75th percentiles: 0.00, 3.00). At day 8, median serum white blood count (WBC) was also notably higher at 24.86 × 10^3^/μL (25th, 75th percentiles: 21.82, 26.90) after MARV/Ang infection (n = 6) compared to 7.07 × 10^3^/μL (25th, 75th percentiles: 4.54, 8.68) after MARV/MtE-Mus infection (n = 17). Also, at day 8, median hemoglobin was lower at 9.75 mg/dL (25th, 75th percentiles: 8.90, 10.60) after MARV/Ang infection (n = 6) compared to 12.50 mg/dL (25th, 75th percentiles: 11.60, 12.90) after MARV/MtE-Mus infection (n = 17).

Elevated CRP (≥2× ULN) was observed at a median of 8 days (25th, 75th percentiles: 6, 9) and preceded that of WBC elevation which occurred at a median of 10 days (25th, 75th percentiles: 9, 11) (Table 2). The development of anemia (hemoglobin ≤ LLN) also occurred at a median of 6 days (25th, 75th percentiles: 4, 10). MARV/Ang was associated with a statistically significant increased risk of CRP elevation compared to MARV/MtE-Mus (HR = 22.44; 95% CI: 3.34, 150.87) (Table 3). MARV/Ang was also associated with statistically significant increased risk of leukocytosis compared to MARV/MtE-Mus (HR = 10.48; 95% CI: 2.39, 33.95). Though CRP and WBC elevation occurred earlier after MARV/Ang versus MARV/MtE-Mus inoculation, no difference was observed in the risk of development of anemia (HR = 1.51; 95% CI: 0.55, 4.18).

#### 3.4.2. Moderate-to-Severe Organ Dysfunction

Median organ markers were plotted by variant over time (Figure 3). Although laboratory values were not available from every day post-exposure, days with measurements which included both variants afforded direct comparisons at different times post-infection. On day 4 post-exposure, the median serum AST was higher during MARV/Ang infection (n = 6) at 81.50 U/L (25th, 75th percentiles: 77.00, 89.00) than at 43.00 U/L (25th, 75th percentiles: 39.00, 49.00) after MARV/MtE-Mus infection (n = 6). Median serum ALT was similar at day 4 at 51.50 U/L (25th, 75th percentiles: 43.00, 62.00) after MARV/Ang infection (n = 6) to that of 57.50 U/L (25th, 75th percentiles: 46.00, 74.00) after MARV/MtE-Mus infection (n = 6). However, by day 8, median serum ALT was higher during MARV/Ang infection (n = 6) at 781.50 U/L (25th, 75th percentiles: 420.00, 1220.00) than at 354.00 U/L (25th, 75th percentiles: 180.00, 502.00) after MARV/MtE-Mus infection (n = 6). Additionally, on day 8 post-exposure, median serum creatinine was notably higher at 3.25 mL/minute (25th, 75th percentiles: 2.60, 4.60) after MARV/Ang infection (n = 6) compared to 1.00 mL/minute (25th, 75th percentiles: 0.90, 1.10) after MARV/MtE-Mus infection (n = 17). 

The risk of liver transaminase elevation after MARV/Ang infection compared to MARV/MtE-Mus infection was significantly higher with an adjusted HR of 2.97 for AST elevation (95% CI: 1.27, 6.91) and an adjusted HR of 3.41 for ALT elevation (95% CI: 1.39, 8.37). There was also a statistically increased risk for creatinine elevation of 43.40 (95% CI: 4.93, 382.04) between MARV/Ang and MARV/MtE-Mus infection.

#### 3.4.3. Coagulopathy

**Figure 4:**
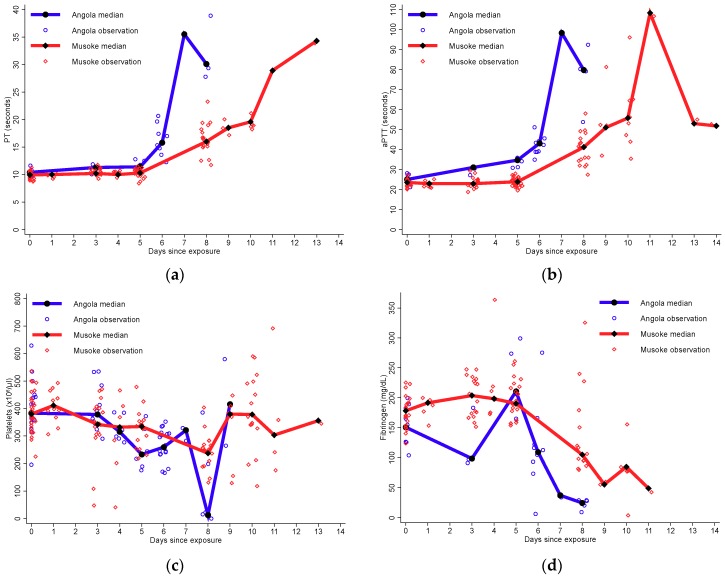
Median and individual coagulation values over time by variant for (**a**) prothrombin time (seconds); (**b**) activated partial thromboplastin time (seconds); (**c**) platelet count (platelets per microliter of blood); and (**d**) fibrinogen (mg/dL).

Similar to organ function and measures of immune activation, the comparisons between the two variants were limited due to heterogeneity in data collection (Figure 4). The largest difference overall for median coagulation parameters between the two variants was observed on day 8. After MARV/Ang infection, median aPTT was higher on day 8 at 79.75 seconds (25th, 75th percentiles: 67.15, 86.55) than at 41.20 seconds (25th, 75th percentiles: 34.80, 44.20) after MARV/MtE-Mus infection. After MARV/Ang infection, the median PT was also higher on day 8 at 30.10 seconds (25th, 75th percentiles: 28.60, 38.70) than at 16.00 seconds (25th, 75th percentiles: 14.80, 18.40) after MARV/MtE-Mus infection. Median fibrinogen was decreased at 24.60 mg/dL (25th, 75th percentiles: 22.50, 27.50) after MARV/Ang virus infection compared to 105.00 mg/dL (25th, 75th percentiles: 91.90, 175.00) after MARV/MtE-Mus infection. Lastly, platelet count was decreased to 12.85 × 10^6^/μL (25th, 75th percentiles: 10.50, 209.00) after MARV/Ang infection compared to 237.00 × 10^6^/μL (25th, 75th percentiles: 200.00, 259.00) after MARV/MtE-Mus infection. 

In our time to event analysis, when evaluating all inoculated NHPs, a decrease in platelets (≤LLN), a decrease in fibrinogen (≤LLN), and an increase in aPTT (≥2× ULN) were observed at median day 8. PT elevation (≥2× ULN) was observed later at day 11 (25th, 75th percentiles: 10, 13) (Table 2). Laboratory abnormalities of clotting and coagulation were observed earlier in the MARV/Ang exposed group compared to the MARV/MtE-Mus exposed group. 

An overall increased risk of aPTT, PT, or fibrinogen abnormalities was observed during MARV/Ang infection compared to MARV/MtE-Mus infection. After the adjustment of baseline covariates, MARV/Ang was associated with an increased risk of aPTT elevation (HR 5.46; 95% CI: 1.73, 17.21) and PT (HR 20.30; 95% CI: 2.03, 203.02). MARV/Ang was associated with a statistically significant increased risk of fibrinogen decrease (HR 7.37; 95% CI: 2.07, 26.28) but not thrombocytopenia (HR 2.60; 95% CI: 0.95, 7.16).

## 4. Discussion

This study, using the largest Marburg virus-exposed NHP data set to date, demonstrates that disease and death occur earlier in NHPs after MARV/Ang exposure than MARV/MtE-Mus exposure. Clinical signs and laboratory values consistently demonstrated earlier signs of disease during MARV/Ang infection. The observed earlier time to death following MARV/Ang was consistent with a previous study that compared four NHPs exposed to the MARV/Ang virus to 11 historical NHPs exposed to the MARV/MtE-Mus [17]. Strengths of the present study include the pooled nature of the data collected under the same fixed conditions and setting, route of entry, and target dose. Despite these significant findings, there are still questions that remain pertaining to the mechanism of accelerated disease and the generalizability to human disease.

It is not clear why a more rapidly fatal clinical phenotype would arise from MARV/Ang infection compared to the MARV/MtE-Mus. Prior research has provided putative biological grounds for this observation. We considered three potential mechanisms. First, differences in the VP40 and L genes have been noted between the guinea pig lethal and nonlethal MARV/MtE-Mus suggesting that key changes in only a few genes could lead to a more virulent phenotype [33]. Further evaluation of the association between genotypic differences and phenotype for MARV variants should be performed as it would provide valuable evidence to support a mechanism behind the increased virulence of MARV/Ang, as well as reveal potential novel therapeutic targets.

The second potential mechanism of increased virulence is that of a higher rate of replication of MARV/Ang compared to the MARV/MtE-Mus. This has been demonstrated in vitro but not demonstrated in vivo previously [26]. It was hypothesized that increased replication could be from observed sequence variation in non-coding regulatory regions [26]. Furthermore, research has suggested more efficient entry into hepatocytes and the reticuloendothelial system by C-type lectin-mediated entry by MARV/Ang compared to the MARV/MtE-Mus [27]. These effects appeared to be due to a single amino acid mutation. One could surmise that more efficient entry could lead to earlier viremia and seeding of virus into the organ parenchyma leading to earlier multiorgan failure and death. A peak viral load was observed earlier during MARV/Ang infection in our analysis. Early virus detection after MARV/Ang exposure was not statistically significantly different to that after MARV/MtE-Mus exposure; however, a higher risk of high viremia was noted using regression, which provides support for an increased level of dissemination. Early dissemination of virus may be a factor of the increased virulence of the Angola variant, but requires further study.

The third potential mechanism of less accelerated disease after MARV/MtE-Mus exposure could be due to the attenuation of virus from multiple passages in tissue culture. The Musoke isolate underwent six passages in Vero cells while Angola isolate had three passages. Therefore, it is possible that viral adaptation in tissue culture could have led to chance genetic changes, leading to slower onset of disease and death in NHPs [14]. Accordingly, the Musoke isolate could be more attenuated than naturally circulating virus which could affect the generalizability of NHP studies that have used this isolate. A direct comparison between the nucleotide sequence between the MARV/MtE-Mus used in NHP studies and the earliest nucleotide sequence with a smaller passage history could be performed [11]. Future research could help to determine if attenuation is a potential mechanism of slower onset of disease. 

There are several limitations to the present investigation. Although increased statistical power was achieved from the aggregation of data from multiple protocols, this led to heterogeneity of the experimental study primates and data collection procedures. Specifically, age was significantly higher among NHPs in studies that used MARV/Ang. However, adjusting for age did not change the interpretation of results of the primary and secondary outcomes. Given the heterogeneity in laboratory data collection between included protocols, our results should be interpreted as exploratory in nature, though suggestive of differences between the two variants. Additionally, the subjectivity and subtle differences in euthanasia protocols could have introduced misclassification into the outcome of death. This could have led to a bias of unknown direction. Furthermore, fever data was not readily available for most studies at the time of our analysis. Fever has previously been described to occur at 4 days post-exposure [30], earlier than anorexia, which was the first sign observed in our study. Aggregated fever information could provide more information about the incubation period of disease for comparative studies. The standardization of future protocols for both data collection and euthanasia would assist future efforts to aggregate data to explore similar pathophysiological questions.

Perhaps more importantly than differences between variants, both MARV/MtE-Mus and MARV/Ang exposure led to more severe disease in non-human primates than what has been described in humans. The efficiency to which the animal model for Marburg virus disease recapitulates human disease is controversial. This study provides further evidence that there are gaps between the clinical presentation of Marburg virus disease in non-human primates and humans.

The high frequency of loss of appetite and rash observed in this study may be more severe than prior reports of MVD in humans. Although this could be subjective and poorly recorded in human cases, during the Angola outbreak, 66% percent of patients developed anorexia which was lower than the 96% in our NHP results [34]. Additionally, a high proportion (94%) of NHPs developed a rash. This is consistent with the largest case series from the Marburg, Germany outbreak in 1967. All 23 well-documented cases had a morbilliform rash. However, rash was not reported from a description of 41 cases from the Angola outbreak or the two cases from the 1980 Kenya outbreak [13,34]. It is possible that a rash occurred but was not documented or it was not noticed due to dark skin pigmentation. In general, our findings suggest a higher proportion of NHPs developed clinical signs of anorexia and rash than in human disease. 

Laboratory findings of transaminase elevation, thrombocytopenia, leukocytosis, and coagulopathy were generally consistent with what has been described in human cases [35]. However, only laboratory results from one of the two cases from the 1980 Kenyan outbreak is available in the literature and there are no published laboratory findings available from the 2004-2005 Uige, Angola outbreak to our knowledge. The one fatal 1980 Kenya outbreak case had an ALT level of 3000 U/L and AST of 6000 U/L, results at the upper limit of detection, and gross pathology was consistent with fulminant hepatic necrosis [13]. More human clinical data would be needed to directly compare our laboratory results in NHPs to human cases. 

This study provides evidence that both variants result in faster and more fatal disease in the cynomolgus animal model than in humans. In our study, Marburg virus intramuscular exposure was 100% fatal compared to a 54% mortality in a meta-analysis of human cases [3]. Additionally, 100% mortality has also been noted previously with lower doses via the intramusular or aerosol routes of MARV/Ang exposure [24]. While the survival rate from the 1980 Kenya outbreak is too low (1 out of 2) to directly compare to the 0% NHP survival, 12% of patients from the 2004–2005 Angola outbreak survived [7,13]. Additionally, our data provide evidence that the course of disease in NHPs is faster than in humans. The time from onset of illness to death has previously been described as a median of 9 days in humans with an incubation period ranging from 5 to 13 days [4,36]. Yet, the incubation period and time from onset of illness to death combined was a median of 9 days in our NHP results. A more rapid onset of disease is also observed in the NHP model of Ebola virus disease compared to human disease [37]. A method of exposing NHPs to Ebola virus intranasally has recently been developed, which may more accurately represent the more prolonged disease course in humans [38]. Further work could evaluate the disease course after intranasal exposure of Marburg virus disease in NHPs. Ultimately, however, more clinical data in humans will be needed to perform more rigorous comparisons to make definitive conclusions about the ability of the NHP model to recapitulate human disease [39].

## Figures and Tables

**Table 1:** Characteristics of cynomolgus macaques and Marburg virus exposure at baseline, overall and by variant type.

Characteristic	Overall	MARV/Ang	MARV/MtE-Mus	*p*-value
	(N = 47)	(N = 18; 38.3%)	(N = 29; 61.7%)	
Sex: no. (%)				
Male	25 (53.2)	9 (50.0)	16 (55.2)	0.737
Female	22 (46.8)	9 (50.0)	13 (44.8)	
Age (years), mean (SD)	5.63 (1.07)	6.14 (1.12)	5.31 (0.91)	0.008
Weight (kg), mean (SD)	4.93 (1.40)	5.12 (1.66)	4.81 (1.22)	0.456

SD, standard deviation; kg, kilograms.

**Table 2:** Time to outcome for cynomolgus macaques after Marburg virus exposure, overall and by variant.

Outcome	Total	Overall	MARV/Ang	MARV/MtE-Mus
	No. (%)	Median days (25th, 75th percentiles)	Median days (25th, 75th percentiles)	Median days (25th, 75th percentiles)
Death	47 (100)	9.00 (9.00, 10.00)	8.13 (8.00, 9.00)	9.96 (9.00, 11.00)
Anorexia	45 (96)	7.00 (6.00, 8.00)	6.00 (6.00, 7.00)	8.00 (8.00, 9.00)
Rash	44 (94)	9.00 (7.00, 9.00)	7.00 (6.00, 7.00)	9.00 (9.00, 10.00)
Viremia (N = 41; ≥5.12 log copies/mL)	41 (100)	6.00 (4.00, 6.00)	4.00 (3.00, 6.00)	6.00 (5.00, 8.00)
High viremia (N = 41; ≥6.34 log copies/mL)	39 (95.1)	6.00 (5.00, 8.00)	6.00 (4.00, 6.00)	8.00 (8.00, 8.00)
Anemia * (N = 34)	26 (76.5)	6.00 (4.00, 10.00)	7.00 (4.00, 8.00)	5.00 (4.00, 10.00)
Immune activation				
CRP elevation ^†^ (N = 26)	22 (84.6)	8.00 (6.00, 9.00)	4.00 (4.00, 4.00)	8.00 (8.00, 9.00)
2. Leukocytosis ^†^ (N = 47)	19 (40.4)	10.00 (9.00, 11.00)	8.00 (8.00, 9.00)	10.00 (10.00, ^‡^)
Organ dysfunction				
1. ALT elevation ^†^ (N = 47)	39 (83.0)	8.00 (6.00, 8.00)	6.00 (6.00, 8.00)	9.00 (8.00, 9.00)
2. AST elevation ^†^ (N = 47)	41 (87.2)	6.00 (5.00, 8.00)	5.00 (3.00, 6.00)	8.00 (5.00, 8.00)
3. Creatinine elevation ^†^ (N = 47)	18 (38.3)	10.00 (9.00, 11.00)	8.00 (8.00, 8.00)	11.00 (10.00, 13.00)
Coagulopathy				
1. PT elevation ^†^ (N = 39)	10 (25.6)	11.00 (10.00, 13.00)	8.00 (8.00, 8.00)	11.00 (10.00, 13.00)
2. aPTT elevation ^†^ (N = 36)	22 (61.1)	8.00 (8.00, 10.00)	6.00 (6.00, 8.00)	9.00 (8.00, 10.00)
3. Fibrinogen depression * (N = 39)	22 (56.4)	8.00 (8.00, 10.00)	6.00 (6.00, 8.00)	9.00 (8.00, 10.00)
4. Thrombocytopenia * (N = 41)	23 (56.1)	8.00 (5.00, ^‡^)	6.00 (5.00, ^‡^)	8.00 (5.00, ^‡^)

ALT, alanine transaminase; aPTT, activated partial thromboplastin time; AST, aspartate transaminase; CRP, C-reactive protein; PT, prothrombin time. * ≤ Lower limit of normal for each sex; ^†^ ≥ 2× Upper limit of normal for each sex; ^‡^ 75th percentile not observed.

**Table 3:** Cox proportional analyses of time to outcome, viremia and laboratory abnormalities comparing MARV/Ang to MARV/MtE-Mus.

Outcome	Crude HR (95% CI)	Adjusted HR (95% CI)
Death	22.10 (7.08, 68.93) *	25.08 (18.02, 34.91) *
Anorexia	5.42 (2.55, 11.54) *	6.19 (4.94, 7.76) *
Rash	23.79 (6.49, 87.19) *	21.48 (15.04, 30.70) *
Viremia (N = 41; ≥5.12 log copies/mL)	1.70 (0.87, 3.32)	1.51 (0.69, 3.30)
High viremia (N = 41)	4.06 (1.86, 8.86) *	3.75 (1.53, 9.18)
Anemia ^†^ (N = 34)	1.20 (0.51, 2.81)	1.51 (0.55, 4.18)
Immune activation		
1. CRP elevation ^‡^ (N = 26)	23.94 (4.31, 133.04) *	22.44 (3.34, 150.87) *
2. Leukocytosis ^‡^ (N = 47)	8.98 (2.37, 33.95) *	10.48 (2.39, 46.01) *
Organ dysfunction		
3. ALT elevation ^‡^ (N = 47)	3.15 (1.53, 6.48) *	3.41 (1.39, 8.37)
4. AST elevation ^‡^ (N = 47)	2.62 (1.32, 5.23) *	2.97 (1.27, 6.91)
5. Creatinine elevation^‡^ (N = 47)	40.50 (5.05, 324.82) *	43.40 (4.93, 382.04) *
Coagulopathy		
6. PT elevation ^‡^ (N = 39)	25.17 (2.86, 221.71) *	20.30 (2.03, 203.02)
7. aPTT elevation ^‡^ (N = 39)	5.32 (1.91, 14.79) *	5.46 (1.73, 17.21)
8. Fibrinogen decrease ^†^ (N = 39)	6.72 (2.35, 19.26) *	7.37 (2.07, 26.28) *
9. Thrombocytopenia ^†^ (N = 41)	1.69 (0.72, 3.95)	2.60 (0.95, 7.16)

Note: Hazard ratio adjusted for age, sex, and baseline weight. ALT, alanine transaminase; AST, aspartate transaminase; aPTT, activate partial thromboplastin time; CRP, C-reactive protein; PT, prothrombin time * *p*-value < 0.0036; ^†^ <Lower limit of normal for each sex; ^‡^ ≥2× Upper limit of normal for each sex.

## Supplementary Materials

The following are available online at http://www.mdpi.com/1999-4915/10/11/658/s1, **Table S1.** Laboratory parameter observations by day. **Table S2.** Endpoints for elevation or decrease of different laboratory parameters. For decreasing parameters, endpoints were the lower limit of the confidence interval. Due to right skewed data, endpoints were defined as two times the upper limit of the confidence interval for increasing parameters.
