# Supplementary material for: Virulence of Marburg Virus Angola Compared to Mt. Elgon (Musoke) in Macaques: A Pooled Survival Analysis
Source: Viruses. 2018 Nov 21;10(11):658. doi: 10.3390/v10110658 (PMC6267608; doi:10.3390/v10110658)
Supplement: Supplementary file 1 [file viruses-10-00658-s001.pdf]

Supplementary:

Table S1. Laboratory parameter observations by day.

Parameter	**Variant**	**Day post-exposure***											
		0	1	3	4	5	6	7	8	9	10	11	13
ALT	Angola	18	0	11	6	6	17	10	13	8	8	0	0
	Musoke	29	6	17	6	23	0	0	17	7	12	2	0
aPTT	Angola	10	0	3	0	5	9	1	5	0	0	0	0
	Musoke	24	6	17	0	29	0	0	17	4	8	1	1
AST	Angola	18	0	11	6	6	17	10	13	7	8	0	0
	Musoke	29	6	17	6	23	0	0	17	7	11	1	0
BUN	Angola	18	0	11	6	6	17	10	13	8	8	8	0
	Musoke	29	6	17	6	23	3	0	17	7	16	3	1
Creatinine	Angola	18	0	11	6	6	17	10	13	8	8	8	0
	Musoke	29	6	17	6	23	0	0	17	5	11	2	1
CRP	Angola	10	0	3	6	6	9	2	5	0	0	0	0
	Musoke	29	6	17	6	22	0	0	16	5	14	3	1
Fibrinogen	Angola	10	0	3	0	5	9	1	5	0	0	0	0
	Musoke	24	6	17	6	23	0	0	15	4	6	1	0
Hgb	Angola	18	0	11	6	6	17	10	13	8	8	8	0
	Musoke	29	11	17	6	17	0	0	17	6	14	4	1
Platelets	Angola	18	0	11	6	6	17	10	14	8	8	8	0
	Musoke	29	11	17	6	17	0	0	17	7	15	4	1
PT	Angola	10	0	3	0	5	9	1	3	0	0	0	0
	Musoke	24	6	17	6	23	0	0	17	4	5	1	1
Viral load	Angola	10	0	11	6	6	18	9	8	2	0	0	0
	Musoke	23	0	17	6	11	6	0	17	5	13	3	1
WBC	Angola	18	0	11	6	6	17	11	13	8	8	8	0
	Musoke	29	11	17	6	17	0	0	17	7	15	4	1

ALT, alanine transaminase; AST, aspartate transaminase; aPTT, activated partial thromboplastin time; CRP, C-reactive protein; Hgb, hemoglobin; PT, prothrombin time; WBC, white blood count. *There were no observations on 2 and 12 days post-exposure.

Table S2. Endpoints for elevation or decrease of different laboratory parameters. For decreasing parameters, endpoints were the lower limit of the confidence interval. Due to right skewed data, endpoints were defined as two times the upper limit of the confidence interval for increasing parameters.

Parameter	Male	Female	Abbreviation	Reference
**Immune activation**				
1. **Hemoglobin (mg/dL)**	12.7	10.7	≤ LLN	[31]
2. **CRP (mg/L)**	3.7	3.5	≥ 2x ULN	[32]
3. **WBC (x10^3/µl)**	21.6	26.2	≥ 2x ULN	[31]
**Organ dysfunction**				
4. **ALT (U/L)**	NA	190	≥ 2x ULN	[31]
5. **AST (U/L)**	NA	116	≥ 2x ULN	[31]
6. **Creatinine (mg/dL)**	NA	2	≥ 2x ULN	[31]
**Coagulopathy**				
7. **Fibrinogen (mg/dL)**	100	100	≤ LLN	[31]
8. **Platelets (x10^6/µl)**	330	221	≤ LLN	[31]
9. **aPTT (s)**	39.1	39.9	≥ 2x ULN	[32]
10. **PT (s)**	19.9	19.7	≥ 2x ULN	[32]

ALT, alanine transaminase; AST, aspartate transaminase; aPTT, activated partial thromboplastin time; CRP, C-reactive protein; Hgb, hemoglobin; PT, prothrombin time; WBC, white blood count; NA, not available; LLN, Lower limit of normal for each sex; ULN, Upper limit of normal for each sex.
